# Lucky morning glory, *Calystegia felix* (Convolvulaceae): a new species from Southern California, with notes on the historical ecology of the Chino ciénega belt

**DOI:** 10.3897/phytokeys.32.6020

**Published:** 2013-12-18

**Authors:** Mitchell C. Provance, Andrew C. Sanders

**Affiliations:** 1 UCR herbarium, Department of Botany and Plant Sciences, College of Natural and Agricultural Sciences, University of California, Riverside, California, 92521-0124, USA

**Keywords:** alkali meadow, *Calystegia felix*, Chino Basin, ciénega, Convolvulaceae, new species, seep, Southern California, spring, urban landscape, wet meadows

## Abstract

A new morning glory, *Calystegia felix*Provance & A.C. Sanders **sp. nov.** (Convolvulaceae), is described from the Los Angeles, San Gabriel, and Santa Ana River basins. Historical collections of the species, which prior to 2011 had not been seen alive in 94 years, have been misidentified as *Calystegia sepium* (L.) R. Br. subsp. *binghamiae* (Greene) Brummitt. The undescribed species was rediscovered in the City of Chino in April of 2011, a few miles north of the location where the most recent previous collection had been made by I. M. Johnston in 1917. The plants were found just prior to their likely destruction by grading and trenching for an underground power line. Intensive searches have resulted in the discovery of five additional occurrences, all of them in the City of Chino. *Calystegia felix* is at high risk of soon becoming extinct in the wild. All of the known extant occurrences are associated with well-watered landscaping on recently completed industrial, commercial, and residential developments. Every known living occurrence is within the limits of a ciénega belt, which is now mostly historical. Otherwise, the new species is only known only from collections made around the turn of the 20^th^ century in what are now heavily urbanized areas, including one from South Los Angeles and another from Pico Rivera in Los Angeles County. *Calystegia felix* lacks the large bracts that immediately subtend, and enclose the calyx, which are always present in members of the *Calystegia sepium* complex. Affinities to *Calystegia felix* are found among other western US species with graduated sepals and small, often somewhat remote bracts. We discuss the enduring confusion between *Calystegia felix* and *Calystegia sepium* subsp. *binghamiae*, and differentiate the new species from some of its more likely relatives. The taxonomic treatment is supplemented by photos of herbarium specimens and living plants. We also discuss the ecological setting of Chino’s ciénega belt, which was a mosaic of palustrine wetlands.

## Introduction

*Calystegia* R.Br. is a genus of about twenty-five species, having a worldwide distribution in temperate zones. Its center of diversity is California, where twelve native species, and thirteen additional native subtaxa, are recognized in the latest Jepson Manual (Brummitt 2012). Although similar to *Convolvulus* L., several characters have been used to differentiate *Calystegia* from *Convolvulus*, including pantoporate pollen, oblong stigma pairs with blunt apices, and a unilocular ovary (Lewis and Oliver 1965). Despite the apparent morphological differences, molecular phylogenetic studies in 2002 (Stefanovic et al. 2002) and 2007 (Carine et al. 2007) suggested that *Calystegia* was nested within *Convolvulus*, making *Convolvulus* paraphyletic with respect to *Calystegia*. However, the studies supported a monophyletic *Calystegia*, but sampled very few members of the genus. Furthermore, the studies did not include species with graduated sepals and small, remote bracts.

In 2011, the first author discovered a morning glory in Chino, California, that could not be differentiated from collections determined by the late R. K. Brummitt as *Calystegia sepium* (L.) R. Br. subsp. *binghamiae* (Greene) Brummitt, or “Calystegia *binghamiae*” (an unpublished name that he had written on one of the specimens years before). Until rediscovered in 2011, this taxon had been widely considered extinct (Brummitt 2012), as the most recently collected specimen authenticated by Brummitt was a specimen collected at Chino Creek in 1917 (*I.M. Johnston 1274*). The site of the rediscovery was along a public walkway in a Southern California Edison (SCE) right-of-way in the City of Chino, roughly four to five miles north of Johnston’s collection locality. This morning glory was confined to an irrigated open-bottom planter at ground level on native soil.

The rediscovery was followed by elevation of *Calystegia sepium* subsp. *binghamiae* to species rank under *Calystegia* (Brummitt et al. 2012) because a reevaluation of the material indicated to them that the taxon warranted species rank. Commendably, their paper drew attention to the astonishing reappearance of a rare taxon in Chino that had been unseen for 94 years. It also completed the lectotypification of *Convolvulus binghamiae* Greene, which had been initiated by Jepson (1939) when he made an earlier combination, *Convolvulus sepium* var. *binghamiae* (Greene) Jepson. Brummitt et al. (2012) noted that more eastern specimens of *Calystegia binghamiae* had more linear to narrowly elliptic bracteoles than were observed in western and more northern populations, and that sepals were at least sometimes inserted significantly below the sepals. They also recognized differences in leaf characters, particularly with regard to basal lobe size and shape. After having received both the lectotype and high-resolution digital images of all of the other known original material (except an isolectotype putatively held at F) for closer examination, we are now certain that the Chino material represents a distinct taxon that does not bear a close relationship to *Convolvulus binghamiae* Greene.

The six recent *Calystegia* locations from Chino are a new species. This new species is also known from three historical collections, including one each from South Los Angeles, Pico Rivera, and Chino. *Calystegia felix* Provance & A.C. Sanders, sp. nov., lacks the large bracts that immediately subtend, and clasp the calyx, as are always present in members of the *Calystegia sepium* complex (Brown et al. 2009). We suspect that *Calystegia felix* is more closely related to species of *Calystegia* endemic to the western US that possess graduated sepals, and have relatively small and sometimes remote bracts. Based on data gained from personal observations, herbarium collections, the early literature, and old maps, we think that *Calystegia felix* is restricted to ciénega-wetland complexes in Southern California’s alluvial basins. The extant occurrences in Chino must represent either plants that germinated from latent seed banks or are resprouts from the roots of plants that persisted in fields when this was an agricultural area. Either way, they apparently reappeared following the introduction of landscaping practices that have brought about “moist ground” conditions, similar to those that were historically present.

## Methods

Recent *Calystegia* specimens from Chino were compared with *Calystegia* and *Convolvulus* collections at RSA-POM and UCR, as well as a selection of specimens from CAS and UC-JEPS, including the lectotype of *Convolvulus binghamiae*. We also obtained high-resolution digital images of *Convolvulus binghamiae* original material held at G-NDG. Scores of *Convolvulus* and *Calystegia* specimen images were evaluated for their relevance to the present study. We obtained images of relevant specimens held at the following herbaria: CAS, DS, E, K, LSU, NA, NY, P, SOC, UC-JEPS, US and WWB. Specimens with little immediate relevance are not listed in the appendices; however, regardless of ultimate relevance, all image sources and their source herbaria, are provided in Appendix I. *Calystegia felix* specimens that were examined are cited in the taxonomic discussion. All specimens of *Calystegia sepium* examined are cited in Appendix II. Species of *Calystegia* that we think could be most easily confused with *Calystegia felix* are compared across a number of characters (Table 1). Specimens that we examined of the species included in that table are cited in Appendix III. Clarifications added to specimen citations appear in brackets. The precisions of the reported geographical coordinates were reduced to ± 300 m for the *Calystegia felix* collections. Only specimens and specimen images that were seen by the authors are cited in the appendices, except in the case of a putative sheet of *Convolvulus binghamiae* at F (*Mrs. R.F. Bingham s.n*.) cited in the original description, and one specimen of *Calystegia felix* (*J.M. Wood et al. 4092*). The herbarium code is followed by the word “image” when only an image was examined. Measurements were obtained from specimens conventionally, or from images using Meander V 2.3 (Dixon and Coventry 2008, available at http://www.fastforwardsw .com/products/meander/). Cultivated plants were grown in UC mix outdoors in partial shade, at about 250 m elevation, in Riverside, California. The historical ecological setting in Chino was compiled from historical maps, early literature, herbarium specimens, and field observations.

**Table 1.** A comparison of floral and vegetative structures in *Calystegia felix* with four similar species of *Calystegia*.

**Character**	*Calystegia felix* Provance & A.C. Sanders	*Calystegia occidentalis* (A.Gray) Brummitt subsp. *occidentalis*	*Calystegia occidentalis* subsp. *fulcrata* (A.Gray) Brummitt	*Calystegia peirsonii* (Abrams) Brummitt	*Calystegia subacaulis* subsp. *episcopalis* Brummitt
habit	clambering to climbing	clambering to climbing	trailing to clambering	tangling subshrub	decumbent to trailing
number of flowers per inflorescence	1, or rarely 2–4	1–4	1	1–2	1
corolla tube width, most proximal visible point (mm)	4–5.9	6–9	5	5.2–6.5	3.3–5.7
sepal shape	narrowly oblong	oblong to oblong-ovate	lanceovate to obovate	oblong to oblong ovate	lorate to narrowly lanceolate
sepal width (mm)	2.5–5	6–9	4–6	57	2–4
ovary internal vestiture	glabrous	silky hairy	silky hairy	silky hairy	glabrous
ovary external vestiture	glabrous or obscurely minutely pubescent apically	subglabrous	glabrous	glabrous	glabrous
inflorescence bract length (mm)	5–14	4–16	6.5–18	5–8	5.5–18.5
inflorescence bract shape	entire; narrowly elliptic to obelliptic	entire; narrowly elliptic to elliptic	lobed; triangular-hastate	entire; ovate, oval, or elliptic	entire; ovate to narrowly elliptic
lamina subtending flower, main lobe shape	broadly ovate to oblong ovate	narrowly triangular to broadly ovate	narrowly triangular to ovate	linear lanceolate to triangular	lance-ovate to broadly ovate
lamina subtending flower, basal lobe shape	short, rounded to truncate	short or not, 2-lobed to bipartite,	long, acute to truncate	long	short, somewhat rounded to narrowly lanceolate
lamina subtending flower, basal lobe orientation	barely divergent to parallel	divergent	divergent to parallel	divergent to parallel	strongly divergent
lamina subtending flower, length along midrib (mm)	45–122	32–51	27–46	14–16	23–28
lamina subtending flower, greatest width (mm)	30–96	33–66	35–54	19–22	16–34
lamina subtending flower, margin contour	flat to laxly involvute, or with inverted basal lobes	flat	flat, or sometimes slightly wavy (esp. in the San Gabriel Mtns.)	wavy to grotesquely curled, rarely flat	flat

## Taxonomic treatment

### Calystegia felix

Provance & A.C. Sanders sp. nov.

urn:lsid:ipni.org:names:77134775-1

http://species-id.net/wiki/Calystegia_felix

Fig. 1

#### Diagnosis.

Differs from *Calystegia subacaulis* Hook. & Arn. subsp. *episcopalis* Brummitt, by its clambering to strongly climbing stems (versus decumbent to trailing stems in *Calystegia subacaulis* subsp. *episcopalis*), larger leaves, 45–122 mm long, 30–96 mm wide mm long, subtending the peduncle (versus 23–28 mm long, 16–34 mm wide in *Calystegia subacaulis* subsp. *episcopalis*), with short, rounded, barely divergent to parallel basal lobes, or sometimes nearly without basal lobes, and essentially truncate (versus the basal lobes somewhat rounded to narrowly lanceolate and strongly divergent in *Calystegia subacaulis* subsp. *episcopalis*); Differs from *Calystegia occidentalis* (Gray) Brummitt subsp. *occidentalis* by its narrowly oblong, 2.5–5 mm wide sepals (versus oblong to oblong-ovate, 6–9 mm wide sepals in *Calystegia occidentalis* subsp. *occidentalis*), narrower corolla tube (basally) 4–5.9 mm wide measured at the most proximal visible point (versus 6–9 mm in *Calystegia occidentalis* subsp. *occidentalis*), an ovary that is glabrous on inside walls (versus a silky hairy vestiture inside of the ovary in *Calystegia occidentalis* subsp. *occidentalis*), and larger, 45–122 mm long, 30–96 mm wide, oblong-ovate to broadly ovate leaves subtending the peduncles (versus smaller leaves subtending the peduncle, 32–51 mm long, 33–66 mm wide, narrowly triangular to broadly ovate), and short, rounded, barely divergent to parallel basal lobes, or leaves that are nearly truncate at the base (versus leaves with divergent basal lobes of varying length that are 2-lobed to bipartite).

**Figure 1.**
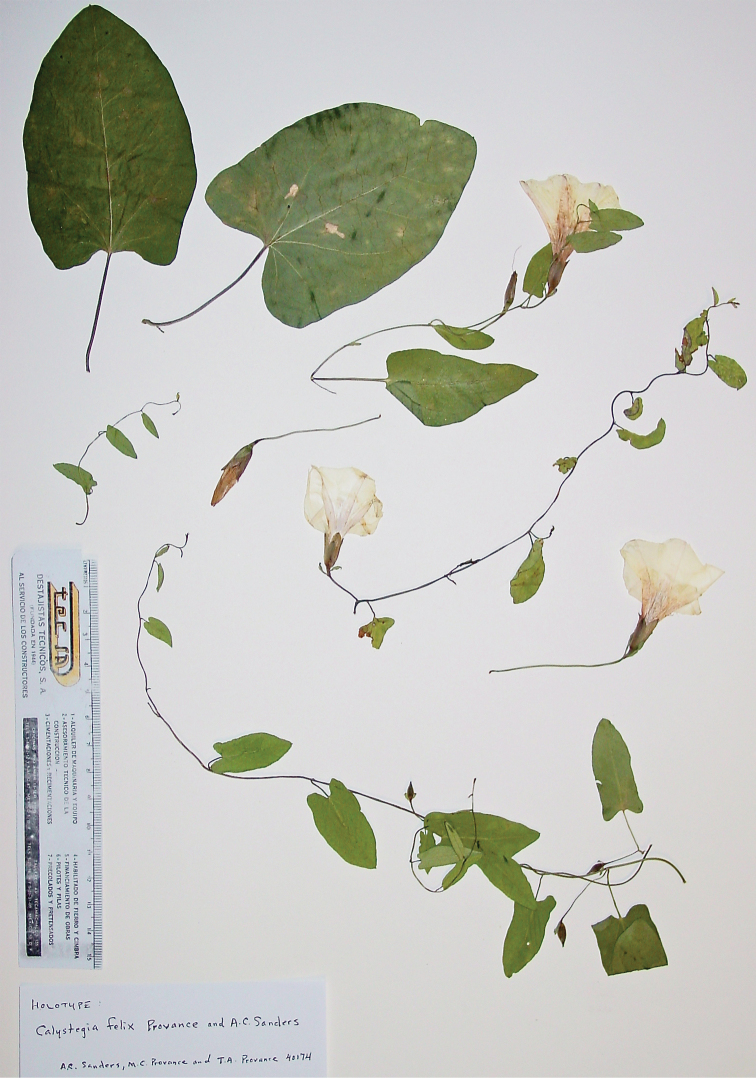
*Calystegia felix* Provance & A.C. Sanders, sp. nov. The holotype, *A.C. Sanders & M.C. and T.A. Provance 40174* (UCR [UCR-246125]). The flowering branchlets are from a single ramet (Photo M. C. Provance, 2011).

#### Type.

USA. California: San Bernardino County, City of Chino, SE of intersection of Edison Ave. and Oaks Ave., edge of Chaffey College Chino Campus, public right-of-way along powerlines. 33°59.822'N, 117°40.518'W, 206 m, 19 May 2012, *A.C. Sanders, M.C. Provance, & T.A. Provance 40174* (holotype: UCR! [UCR-246125]; isotypes: ARIZ!, CAS!, K!, MO!, NDG!, NY!, RSA!, SBBG!, SD!).

#### Description.

**Figure 2.**
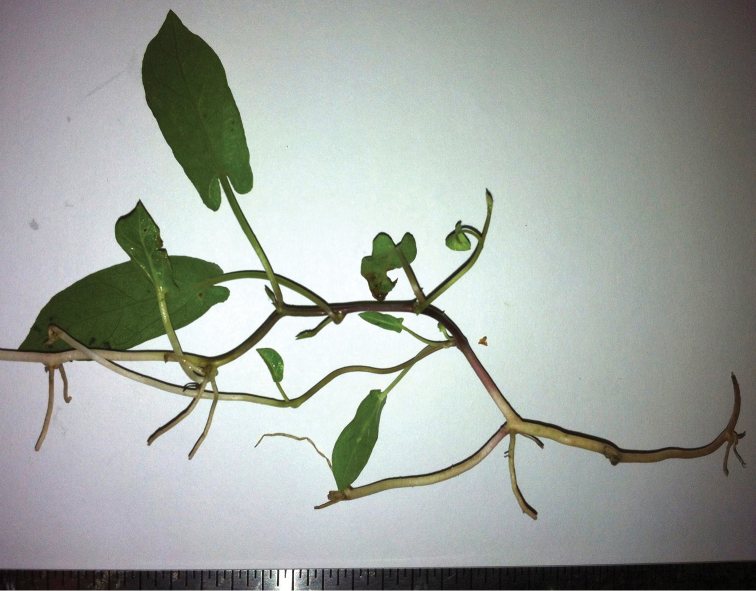
*Calystegia felix* stolons and creeping rootstock. The narrow emergent leaves of this specimen may be atypical; the relatively long petioles are normal (Photo M. C. Provance, 2012).

**Figure 3.**
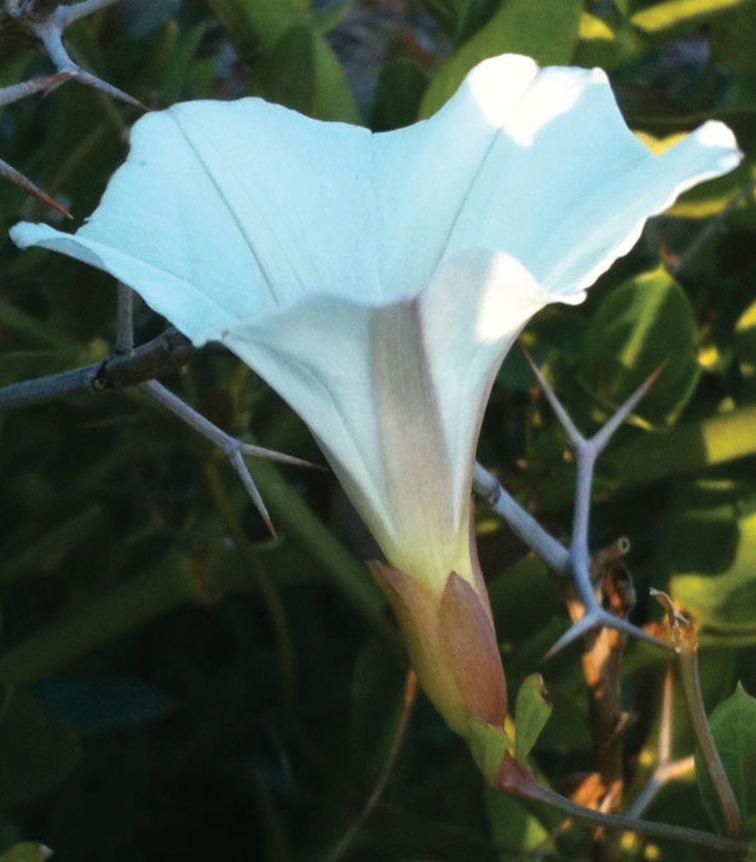
*Calystegia felix* in bloom at the type locality, in a planter bed in Chino. This plant, discovered in 2011, is the only one we have seen that produces corollas with reddish-purple midpetaline stripes. Also, note the blushed sepals (Photo M. C. Provance, 2011).

Semi-herbaceous perennial vines, senescing in October, though with some stems and leaves persisting through winter. Aerial stems 1–3 m long, from shallow, creeping rhizomes and stolons (Fig. 2), climbing and twining, or clambering across shrubs, branching frequently, terete, with nonobvious longitudinal ridges, slender, tough and wiry, glabrous to sparsely hairy, in life dull grayish pink to light green with a rosy cast. Leaves alternate, membranaceous to chartaceus, glabrous to sparsely hairy, bicolored when mature green above, paler below, relatively flat and not folding along the midrib, but sometimes the basal half of the lamina slightly involute, and often having the basal lobes abruptly turned upward. Petioles on climbing stems 0.3–0.5 × length of lamina, e.g. about 14–61 mm long, but often longer relative to lamina length on emergent leaves; lamina of climbing stems 45–115(–122) mm long, 30–80(–96) mm wide, oblong-ovate to broadly ovate, but narrowly oblong on the sterile branchlets and on stems distal to the flowering axils, base cordate, with short, rounded, parallel or barely diverging basal lobes, sometimes essentially without basal lobes and nearly truncate, apex obtusely rounded, sometimes subacute, minutely apiculate; emergent leaves from rhizomes and on trailing stems variable in shape, but usually broadly oblong to oval or orbicular, sagittate, with short lobes, or lobeless and rounded to the petiole, apex broadly rounded; lamina venation obscurely pinnate, but with 2–4 lateral veins from the base. Inflorescences axillary, flowers usually solitary, rarely 2–3(–4)-flowered; pedicels 1–30 mm long, peduncles 18–63 mm long; bracts 2, attached (1–)2–3(–4) mm below the calyx, ascending, subopposite, 5–14 mm long, 1–2.5(–3.5) mm wide, narrowly elliptic to narrowly oblanceolate, obtusely pointed, ± flat, with a raised midvein, glabrous to scantly puberulent. Flowers perfect; sepals 5, entire, graduated, narrowly oblong to lanceovate, green with a rosy blush, short-ciliate, inner sepals 11–15 mm long, 3.5–4 mm wide, the lower portion tightly appressed to mature fruit, outer sepals 8–11 mm long, 2.5–5 mm wide, apices ± acutely rounded, mucronulate; corolla funnelform, 27–45 mm long, base of visible tube 4–5.9 mm wide, white (sometimes appearing light yellow in herbarium specimens), with 5 externally pigmented interplicae (midpetaline bands or longitudinal stripes), these very light-yellow, more rarely reddish-purple (Fig. 3), glabrous externally, or rarely, conspicuously hairy adjacent to pleats in the basal third of the corolla, the hairs yellowish, lobes 5, very short, each with a concentrated area of minute hairs along the margin; stamens 5, equal; filaments 18–21 mm long, fused to the corolla tube ± 7-9 mm of that length, glandular hairy along the proximal margins; anthers 4–4.5 mm long, white, barely reaching the base of the stigmas; pistil glabrous both internally an externally; style 16–21 mm long, glabrous, or with a few glandular hairs near the base; stigmas 2, cylindrical, ± 3 mm long, asymmetric, with one axially oriented, and the other ascending; nectary crenate-coronoid. Pollen white to cream, with circular perforations discernible at 60 X. Fruit dry capsule, indehiscent to tardily dehiscent from tip to base, globose, 9–10 mm in diameter, glabrous or obscurely minutely pubescent apically. Seeds 1–4 per capsule, ca. 4 mm in height and 3.5–4 mm in width, ± angular-ovoid, and depending on the number of developing seeds, nearly black to dark brown and tan-speckled, hilar region purplish, finely granular.

#### Distribution.

**Figure 4.**
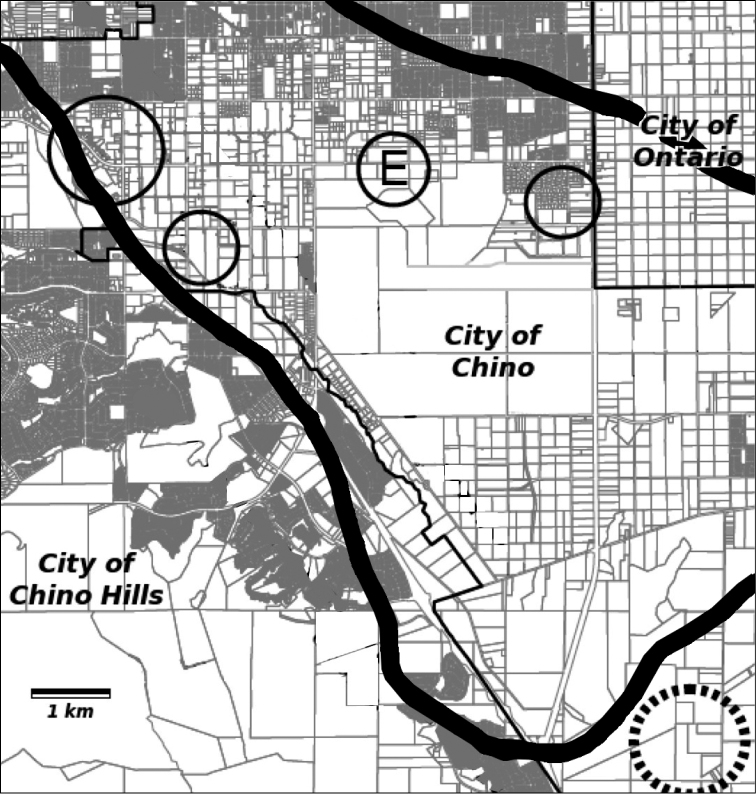
Map of historical and recently discovered *Calystegia felix* occurrences in the Chino Basin. The circled E marks the plant discovered in 2011; normal circles are 2013 occurrences, the largest a cluster of three populations. The broken circle is the approximate location of Johnston’s collection in 1917. The perimeter of the Chino artesian belt and “moist land” prior to 1904 is delineated by the thick black line. The street map was adapted from the City of Chino draft general plan EIR, July, 2010, and the artesian belt is based on Mendenhall (1907).

*Calystegia felix* is endemic to the inland basins of the Los Angeles, San Gabriel, and Santa Ana river watersheds in Southern California, at between 40 and 208 meters elevation. The species has not been seen in Los Angeles County since 1902. Six occurrences are known, all of them in the City of Chino, in San Bernardino County (Fig. 4). The occurrences have a spatial separation ranging from 0.3–2 km. The easternmost occurrence is just west of Euclid Avenue, close to Chino’s border with Ontario and Eastvale. The westernmost occurrences are on alluvial terraces above Chino Creek, coming within several meters of the City of Chino Hills.

#### Phenology.

Flowering begins in late March, and is heavy until early August, with flowering thereafter decreasing through late September. In 2011 and early 2012, inflorescences on the only plants known at that time had solitary flowers. During mid-May of 2012, inflorescences at that site were observed to be two or three-flowered, and rarely solitary. It is not certain whether flower number increased as the season progressed, or if flowers during the later visit were originating on vines of a different genet. Only solitary flowers were seen at the sites discovered in 2013. Ripe seeds have been collected from early June until late October. Fruit with small holes indicative of seed predation by bruchid beetles have been found (Provance, pers. obs.). Small, senesced, nodding, sterile, apetalous flowers, mostly near ground level, have recently been noticed on some plants. We observed similar flowers on herbarium specimens of a few other species of *Calystegia*. It is unknown if these flowers are apetalous developmentally, or if the corollas were lost to insect predation. More in-depth study of this condition is needed.

#### Additional specimens examined.

**USA. California. San Bernardino County**: Chino Creek south of Ontario, climbing in trees, 500 ft., 30 May 1917 (fl), *I.M. Johnston 1274* (RSA, POM, UC); City of Chino, 33°59.823'N, -117°40.537'W, 206 m, SCE right-of-way, just northeast of Chaffey College, southeast corner of the intersection of Edison Rd. & Oaks Ave., 11 May 2011, *M.C. Provance 17214* (UCR); same location, 14 May 2011, *M.C. Provance 17351* (UCR, UC, DAV, NDG); same location, 25 Mar 2012, *M.C., J.M., & T.A. Provance 17430* (UCR, WIS); West Chino, east of Chino Creek, 33°59.17'N, 117°42.38'W (± 300 m), 190 m, planter bed in public parking area, 31 May 2013, *M.C. Provance 17525* (UCR, to be distributed); West Chino, east of Chino Creek, 34°00.18'N, -117°43.31'W (± 300 m), 208 m, planter bed in public parking area, 3 June 2013, *M.C. Provance 17526* (UCR, to be distributed); West Chino, Highway 71 – Grand Avenue ramp, 33°59.44'N, -117°43.30'W (± 300 m), 205 m, Highway 71 and an adjacent planter bed in a public parking area, 11 June 2013, *M.C. Provance 17527* (UCR, to be distributed); West Chino, 33°59.63'N, -117°43.18'W (± 300 m), 200 m, planter bed in a public parking area, 11 June 2013, *M.C. Provance 17528* (UCR, to be distributed); East Chino, planter bed along public sidewalk near southeast corner of Buckeye Street and Fern Avenue, 33°59.65'N, -117°39.24'W (± 300 m), 206 m, 11 June 2013, *M.C. Provance 17529* (UCR, to be distributed); City of Chino, SE corner of Edison Ave. and Oaks Ave., near entrance to Chaffey college campus, ca. 2.5 mi N of Chino Creek (Prado Basin), irrigated landscaped area adjacent to ruderal grasslands, 17 May 2011, *J.M. Wood et al. 4092* (K not seen, RSA not seen). **Los Angeles County:** Rivera [historic town, later part of Pico Rivera, misspelled “Riveria” on Davidson’s herbarium label, and misspelled “Riviera” in Davidson and Moxley 1923], “Common in most grounds [moist grounds—a phrase Davidson often used on herbarium labels]” (Davidson 1909), “Very common on stream banks [either the Rio Hondo, or a small unnamed stream running through McCampbell and Downey Road, c. 0.5 km west of Rivera] at Riviera [Rivera] and on the Los Angeles and San Gabriel Rivers in that vicinity” (Davidson & Moxley 1923), 1 May 1902, *A. Davidson 1892* (RSA [RSA-394817]); Near University Station [historic train station in S. Los Angeles at 43^rd^ St. and Vermont Ave. (Grace 2007)], Los Angeles, 1899, *A. Davidson 2144* (RSA [RSA-394819] [mixed collection with *Calystegia sepium*]).

#### Discussion.

***Taxonomy*:** Although arguments to maintain *Calystegia* have been weakened by recent molecular studies, we describe this new species as such, pending molecular phylogenetic studies that sample more thoroughly across both *Calystegia* and *Convolvulus*. Although their flowers are not similar, it is noteworthy that few vegetative characters seem to separate *Calystegia felix* from the weed *Convolvulus arvensis* L. The only vegetative feature we currently know that can reliably be used to tell these taxa apart is the cross section of the stem, which is angular in *Convolvulus arvensis*, and terete with weak longitudinal ridges in *Calystegia felix*. There may be differences in leaf venation, but that will require additional study. Unfortunately, *Convolvulus arvensis* is abundant throughout the Chino area, and occurs at several of the *Calystegia felix* sites.

**Figure 5.**
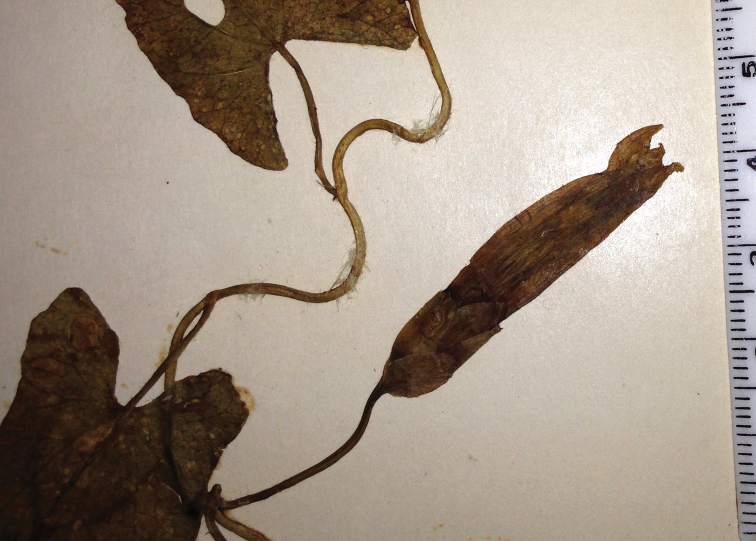
Lectotype of *Convolvulus binghamiae* Greene, Santa Barbara, July, 1886, *R.F. Bingham s.n*. (UC335392), with one small bract, and a broad-based corolla.

**Figure 6.**
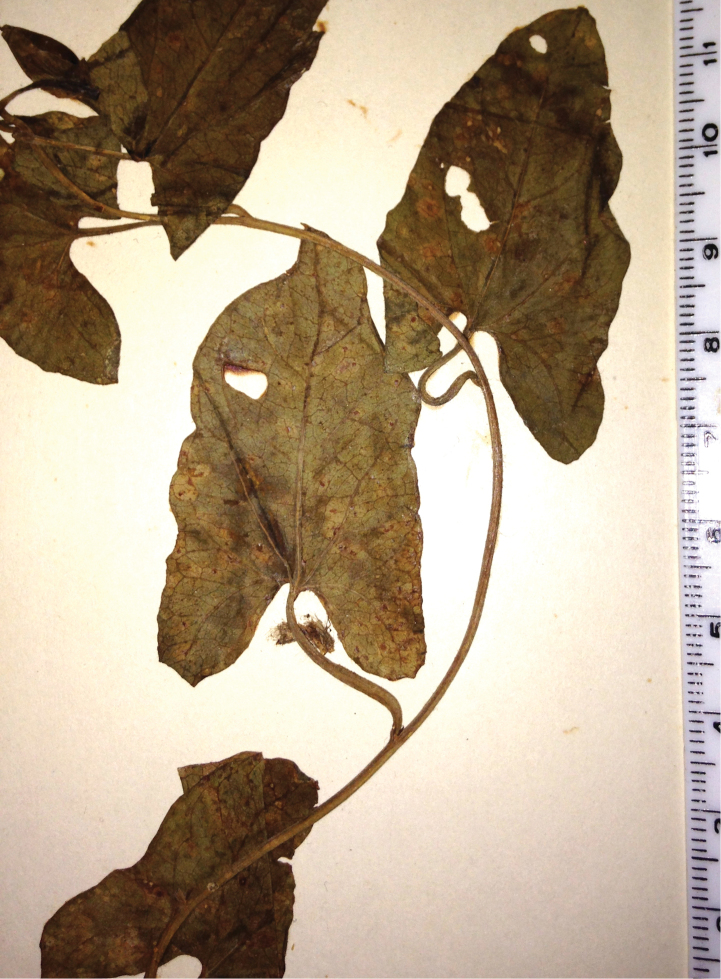
The leaves shown here from *R.F. Bingham s.n*. (UC335392) are consistent with many specimens of *Calystegia sepium*.

**Figure 7.**
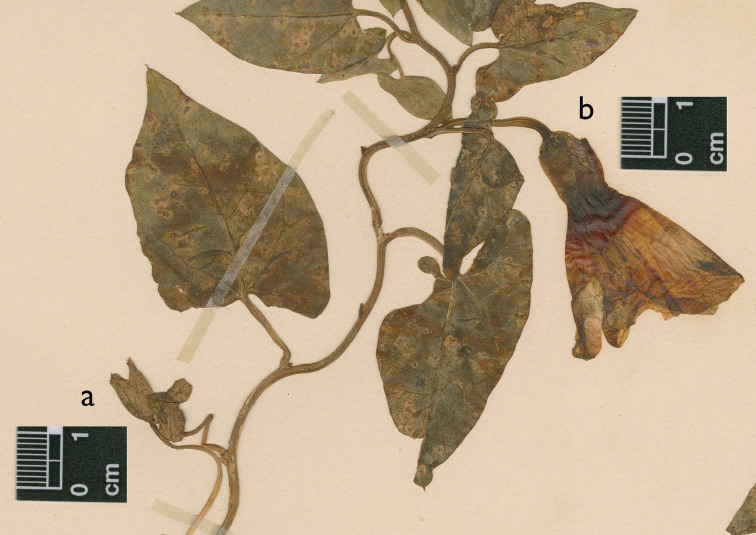
Original material of *Convolvulus binghamiae* Greene, Santa Barbara, 1886, *E.L. Greene s.n*. (NDG [NDG-66275]). Flower buds with large clasping bracts typical of the *Calystegia sepium* complex are noteworthy (a), as is the solitary flower (b) with a broad calyx, broad corolla tube, and a smallish, but otherwise typical clasping bract for the *Calystegia sepium* complex (Image courtesy of Barbara Hellenthal at the Notre Dame Herbarium).

While a definitive treatment of the entire *Calystegia sepium* complex has not been published, the best defining features of this group are the large bracts which immediately subtend, and often enclose the calyx, and have conspicuously netted venation. It is a taxonomically difficult complex that may include over twenty *Calystegia sepium* subtaxa, some additional closely related species, and their subtaxa (Stace 1961, Brown et al. 2009). So defined, all of the original material for *Convolvulus binghamiae*, including the lectotype at UC (Brummitt et al. 2012), is clearly referable to this complex. The epithet *binghamiae* is sometimes applied to specimens from Southern California with clasping bracts that only partly cover the sepals, and have leaves with barely divergent basal lobes, but that are otherwise inseparable from *Calystegia sepium*. All of the original material of *Convolvulus binghamiae* is from a salt marsh that once occurred in Santa Barbara. The lectotype has only one flower (Fig. 5), which has a bract that may be the smallest found on any of the original material. Nonetheless, the corolla has a broad base, as seen in all members of the *sepium* complex. Moreover, leaves from the same sheet (Fig. 5 and Fig. 6) are consistent with many specimens attributable to the *Calystegia sepium* complex. The bracts among the original material range from 7 to 13.8 mm long and 3.1 to 9.1 mm wide, and in their fully developed state are broadly lanceolate to broadly ovate. Inflorescence bracts in *Calystegia felix* have a similar range in length, but are much narrower at 1 to 3.5 mm in width, and usually lack a conspicuous network of veins. Most bracts in the *Convolvulus binghamiae* original material are in every sense typical of the *Calystegia sepium* complex. Interestingly, some of the largest bracts are associated with flower buds: in one case (Fig. 7), some of the bracts of the flower buds are larger than the bracts of the open flower on the same sheet. Finally, the corolla tubes (measured at the base of the sepal lobes) in the *Calystegia binghamiae* original material are over 8 mm wide. In *Calystegia felix* the lower tube of the corolla is narrow, ranging from 4 mm to about 6 mm in width. *Calystegia felix* is clearly not part of the *Calystegia sepium* complex, and represents a new, unrelated, and previously undescribed taxon.

Specimens of *Calystegia felix* were included in *Convolvulus binghamiae* by Davidson in his list of new records for Los Angeles (1909) and by Davidson & Moxley in their flora of Southern California (1923). When Jepson made the combination *Convolvulus sepium* var. *binghamiae* (Greene) Jepson, he was very particular in his application of the name, stating, “Santa Barbara; a distinct localized variety, rarely collected”. Under *Convolvulus sepium* var. *pubescens*, Jepson (1939) cites a Chino specimen (*Condit* s.n.) that we have seen, and which is equivalent to material typically ascribed to *Calystegia sepium* subsp. *limnophila* in Southern California (and elsewhere). While it is possible that Jepson had seen a collection of *Calystegia felix* at some point, it is not obvious where he would have placed such a collection in his 1939 treatment.

In 1945, Abrams annotated one sheet of Johnston’s *Calystegia felix* collection at RSA as *Convolvulus binghamiae*, probably while preparing his Illustrated Flora (1951). The illustration of *Convolvulus binghamiae* in this flora seems to be *Calystegia felix*, which is incongruent with his treatment, since the geographic distribution given by Abrams for *Convolvulus binghamiae* excludes all extant and historic occurrences of *Calystegia felix*. Brummitt (1993) applied *Calystegia sepium* subsp. *binghamiae* (Greene) Brummitt, to plants of the northern and central South Coast between sea level and 20 meters elevation, which excludes collections from Chino. In Brummitt et al. (2012), the author’s recognize the similarity of material we refer to *Calystegia felix* to the illustration in Abrams flora, noting, “A good illustration of the latter may be seen in Abrams (e.g., Fig. 3855, 1951)”. However, they propose that the drawing represents a phenotypic variant of their proposed *Calystegia binghamiae* (Brummitt) Brummitt. The misidentified collections at RSA and the illustration in Abrams of what was actually an undescribed species seems to have influenced the search image of at least some local botanists attempting to rediscover *Calystegia sepium* subsp. *binghamiae*.

Philip A. Munz annotated a Johnston collection at RSA as *Convolvulus purpuratus* Greene in 1931, thus clearly including *Calystegia felix* in his concept of *Convolvulus purpuratus*. In his Southern California Manual (Munz 1935) he listed Chino as a locality for this species. Among many differences, *Calystegia purpurata* (Greene) Brummitt subsp. *purpurata* can be readily separated from *Calystegia felix* by its equal, evenly inserted sepals.

The number of flowers per inflorescence, corolla pigmentation, external corolla vestiture, and the vestiture of leaves and stems vary in *Calystegia felix*. Whether this variation is influenced more by genetics or environmental factors remains to be studied. Heterophylly is profound in *Calystegia felix*, and generally manifests as narrower lamina on sterile twining stems, instead of the larger ovate to oblong-ovate leaves of reproductively active stems. There seems to be a tendency towards rounder leaves with longer petioles on emergent stems and sometimes trailing stems. *Calystegia felix* is similar to other species in the genus with small, somewhat remote bracts, and graduated sepals. Several morphological characters are used to compare four of those species with *Calystegia felix* (Table 1). Leaf parameters alone are often insufficient for the identification of *Calystegia*, but fortunately, several other characters in addition to leaf shape, differentiate *Calystegia felix* from other species of *Calystegia*.

At first glance, *Calystegia felix* looks most similar to *Calystegia occidentalis* (Gray) Brummitt subsp. *occidentalis*, since both taxa have a similar clambering or climbing habit, similar bracts inserted approximately the same distance below the calyx, and potentially produce multiple flowers in inflorescences. However, *Calystegia felix* differs from *Calystegia occidentalis* by its narrower sepals, narrower corolla tube, internally glabrous ovary, and larger oblong-ovate to broadly ovate leaves. The leaves subtending peduncles of *Calystegia felix* have short, rounded, barely diverging to parallel basal lobes. Sometimes, *Calystegia felix* leaves are nearly truncate at the base. This easily differentiates *Calystegia felix* from *Calystegia occidentalis*, which has lamina basal lobes that are of varying length, but divergent, and usually 2-lobed or bipartite. *Calystegia felix* also looks like *Calystegia subacaulis* Hook. & Arn subsp. *episcopalis* Brummitt. Both taxa have slender, but tough and wiry stems, corolla tubes that narrow toward the base, narrow sepals, and an ovary that is glabrous both internally and externally. It differs from *Calystegia subacaulis* subsp. *episcopalis* by its strong climbing habit, and much larger leaves that differ considerably in basal lobe morphology.

The similarities between *Calystegia felix* and *Calystegia subacaulis* subsp. *episcopalis* tend to be less readily apparent than the similarities between *Calystegia felix* and *Calystegia occidentalis* subsp. occidentalis. However, the characters shared seem not to be widespread in the genus. For example, while ovaries of *Calystegia felix* sometimes have a small number of minute hairs toward the apex, they are essentially glabrous externally. They are also glabrous internally. Though we have had only one specimen of *Calystegia subacaulis* subsp. *episcopalis* upon which we have been able to conduct detailed flower dissections (*F. Bowcutt 2163* [UCR]), we are especially intrigued by the ovaries of this collection, which are glabrous both internally and externally. We have seen this combination of characters only in *Calystegia felix*, and similarities such as these might indicate that the two taxa are more closely related than their superficial appearances suggest.

***Ecology*:** The six known occurrences are associated with somewhat poorly drained alkali silt loam (SoilWeb 2013), on a floodplain with an average slope of just over 1% (Lewis Publishing Company 1890, SoilWeb 2013). The local soils have developed primarily from the accumulated granitic alluvium that was washed out of the San Gabriel Mountains during episodic flood events (Hilgard 1902). Historically, there were a number of springs near the *Calystegia felix* occurrences, and the springs of the plains and basins and their accompanying vegetation, typically marshland and wet meadows, were known as *ciénegas* (Schuyler 1880, Mendenhall 1908). In Southern California the use of the word *ciénega* always implied the presence of a spring, unlike in some other parts of the American Southwest (e.g. Hendrickson and Minckley 1985). In the earliest known general description of ciénegas in the Chino Basin, Schuyler (1880) emphasized that *ciénega* was the only word commonly used to indicate its springs and associated habitat. In the Chino Basin, there were two (Hall 1888a, 1888b) or three (Tait 1911) main groups of ciénegas located a very short distance west to southwest, south, and southeast of Chino (Tait 1911). The perimeter of the Chino Artesian Spring Belt was roughly triangular, and its location in the current landscape is easily derived from the early maps. The east and west vertices were near the Chino Creek and Mill Creek emergences respectively. A third vertex would be near the south side of Prado Basin. These boundaries coincide well with the historical limits of “moist land” as mapped earlier by H. B. Martin (1887–1889). Mendenhall (1908) estimated the area of the artesian belt and associated moist soil as 23 sq. miles prior to 1904. Various aspects of the hydrology and geology of the Chino Basin ciénegas have been summarized (e.g. Truman 1874, Schuyler 1880, Hall 1888b, Lewis Publishing Company 1890, Shinn 1898, Mendenhall 1905, 1907, 1908, Hilgard and Loughridge 1906, 1908, Troxell 1957).

Historically, the water table in the vicinity of the artesian spring belt was 6–35 feet below ground (Lewis Publishing Company 1890) The soils within the spring belt, which are largely alkali silt loams, retained moisture throughout much of the year, and as a consequence were extraordinarily important to Southern California agriculture (e.g. Peffer 1894, Shinn 1898, Nelson 1917). Based on soil maps, four of the *Calystegia felix* occurrences are on Chino silt loam. Both of the occurrences that are not on Chino Silt Loam, one on Grangeville fine sandy loam, the other on Hilmar loamy fine sand, are less than 30 feet from Chino silt loam according to soil maps (SoilWeb 2013). While *Calystegia felix* occurrences seem to be strongly associated with Chino silt loam; an analysis of soil at occupied sites has not been performed.

On Edison Rd., *Calystegia felix* was discovered in a sidewalk tree basin on Chino silt loam. In that area, the soil is pale gray, with occasional small patches of fluffy salt crust. Disturbed alkali playa habitat was observed nearby, with *Heliotropium curassivicum* L., *Heliotropium europaeum* L., *Cynodon dactylon* (L.) Pers., *Chenopodium berlandieri* Moq., *Malvella leprosa* (Ortega) Krapov., *Convolvulus arvensis* L., and *Amaranthus palmeri* S. Watson. Also nearby was a sparsely vegetated earth-bottom ditch with *Conyza* and *Lepidium strictum* (S. Watson) Rattan, and old fields with *Secale cereale* and a diverse group of weedy native and introduced forbs. Native plant species documented within 400 m of the *Calystegia* site include: *Amaranthus palmeri*, *Ambrosia psilostachya* DC., *Amsinckia* sp., *Atriplex serenana* Abrams, *Baccharis salicifolia* (Ruiz and Pav.) Pers., *Chenopodium berlandieri*, *Epilobium brachycarpum* C. Presl., *Epilobium ciliatum* Raf., *Fraxinus velutina* Torr., *Pseudognaphalium californicum* (DC.) Anderb., *Heliotropium curassivicum*, *Heterotheca grandiflora* Nutt., *Malacothrix saxatilis* (Nutt.) Torr. & A. Gray, *Malvella leprosa*, and *Solanum americanum* Mill.

**Figure 8.**
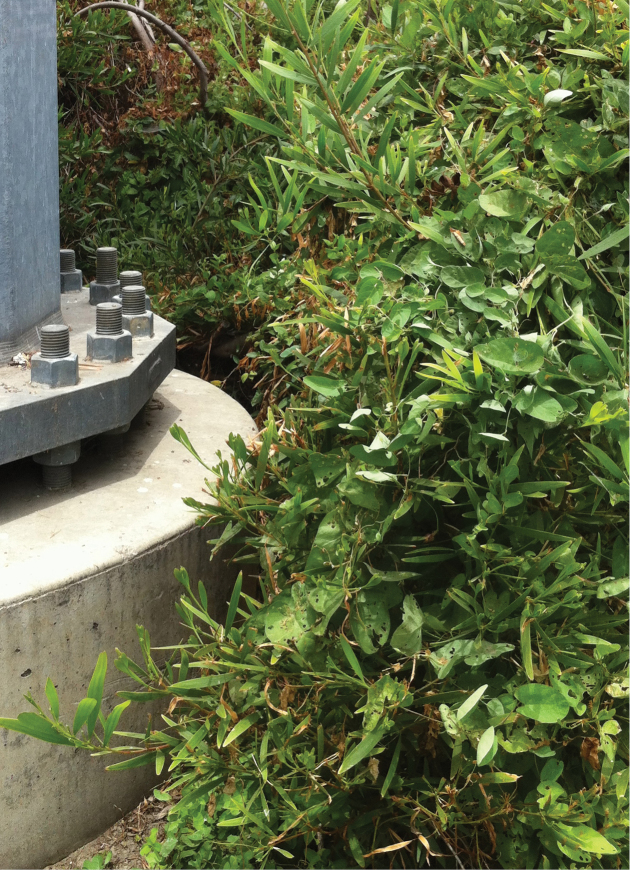
*Calystegia felix* climbing upward through urban landscaping in the City of Chino (Photo M. C. Provance, 2013).

**Figure 9.**
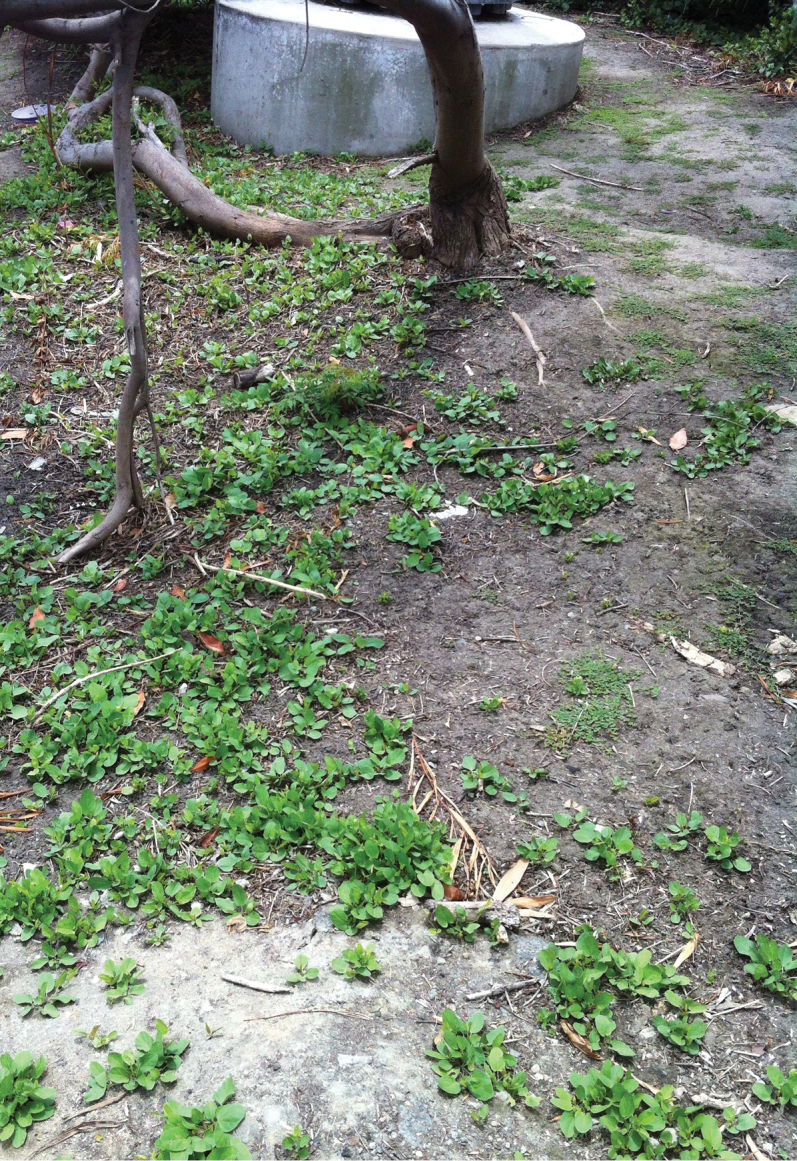
Emergent *Calystegia felix* on crusted, moist, Chino silt loam, amongst urban landscaping. It is unknown if large groups of emergent plants such as these represent one to just a few clones, or many genotypes (Photo M. C. Provance, 2013).

Although seeds and rhizomes can be moved around in many ways, we contend that invoking accidental transport of stem fragments or seed by humans is not the most parsimonious explanation for the presence of *Calystegia felix* in the City of Chino, since the species is known nowhere else. While we have no direct proof, we think the recently discovered *Calystegia felix* populations represent plants that have emerged from latent, long-lived seed banks or roots following a return to “moist soil” conditions (Figs 8, 9), similar to those in the historical record. Buried seeds of *Calystegia sepium* have retained high levels of viability after 39 years (Bond et al. 2007), and *Calystegia felix* may have similar longevity. If changes in soil moisture regimes are occurring (i.e.becoming wetter), horticultural practices within the urban environment are likely the cause. We have not investigated soil moisture in Chino experimentally, but we observed an apparent moisture gradient. The success we have had locating new occurrences of this rare plant in developed areas contrasts sharply with our failure to locate occurrences in undeveloped visually drier areas. While we are not sure of the significance at this point, it seems noteworthy that each of the sites currently supporting *Calystegia felix* were, based on aerial images (Google Earth V.2.1.6014b), completely stripped of their vegetation at some point between 4 and 11 years ago).

Historical information and early herbarium collections suggest that the Chino Basin originally had vegetation of wet meadow and alkali meadows dominated by *Anemopsis californica* (Nutt.) Hook. & Arn., with perennial grasses, such as *Elymus triticoides* Buckley, *Sporobolus airoides* (Torr.) Torr., and *Distichlis spicata* (L.) Greene, and herbs such as *Trifolium willdenovii* Spreng., *Trifolium wormskioldii* Lehm., and *Helianthus annuus* L. In addition, there were small bodies of open water, alkali and freshwater marshes, alkali scrub, alkali grassland, alkali playa, moist stream banks, and willow thickets. There were also phreatophytic woodland communities of *Salix*, *Populus*, and *Platanus racemosa* Nutt. (Truman 1874). The spring-belt wetlands were collectively referred to as “ciénega-lands” (e.g. Hilgard 1889). Common sunflower (*Helianthus annuus*) is frequently mentioned in the early literature as a common species on alkali soils in the Chino Basin, and was considered indigenous (e.g. Davy 1898). The topography just north of the Santa Ana River was reportedly hummocky (Nelson 1917), and may have been supportive of vernal pools. Most fine-scale relief in the basin has probably been lost to disking and grazing cattle, but the north part of the ciénega belt was reportedly rather smooth. Mendenhall (1905) commented:

“*The lands just above this* [above the ciénega-lands] *are flat and often ill drained. The waters rising and evaporating here, under the influence of the effective southern sun, leave behind them their salt content, and thus alkali lands may result*”

Thus, much of the landscape represented a mosaic of ciénega and ciénega-creek associated palustrine communities. The historical natural vegetation of the City of Chino cannot easily be envisioned because of past and current development. For example, a satellite of the University of California Agricultural Experiment Station called the “Ten Acre Tract” used to be in Chino and experiments related to growing crops on alkali soil were conducted there. This property was described as being dominated by *Anemopsis californica* (Hilgard & Loughridge 1896), which indicates that it was likely alkali marsh. The Ten Acre Tract is now occupied by industrial buildings and offices. However, taxa highly indicative of alkaline marsh and alkaline meadow have persisted in unusual places. For instance, we documented a number of *Anemopsis californica* persisting in plantings of *Hedera helix* along a sidewalk in northeast Chino near the Ontario border, just within the mapped historical limits of moist ground. The following year we found *Calystegia felix* growing in a sidewalk planter across the street from the *Anemopsis* site, in similar urban landscaping. We think other remnants of the ciénega flora may persist in Chino.

***Conservation***:* Calystegia felix* is endemic to Southern California, extirpated in Los Angeles County, and now likely confined to the Chino Basin in San Bernardino County. It is doubtless at high risk of soon becoming extinct in the wild. This is due to hydrological changes in the Chino Basin, including the paving of streams, lowering of the water table, and loss of ciénega habitat, including vegetation associated with marshes, meadows, grasslands and alkaline playas; and encroaching commercial, industrial, residential developments, and public works projects. Large areas of habitat have already been transformed. Six extant occurrences are now known, with an estimated 200 ramets emerging in 2013 at a single location near Chino Creek. However, those plants likely represent clones, as do the about 50 ramets at the other sites. Based on there being few known populations, a limited overall distribution, and a small number of individuals in existence, we suggest a conservation status of Critically Endangered (CR). Upon discovering the plants along Edison Road, it was obvious they were in imminent danger of being destroyed by impending grading and trenching for the burial of high-voltage power lines. We initially thought that these plants represented a single clone, but two ramet-specific flower color morphs, seed production, and spatial separation of clusters of emergent stems, suggest that two or more genotypes are present. Over the short term, Rancho Santa Ana Botanical Gardens has been contracted to conduct ex-situ propagation of rhizomes from the Edison Road population, and a few other plants are being cultivated in private and institutional gardens.

## Supplementary Material

XML Treatment for Calystegia felix

## Appendix I

**Table:** Image databases, internet address, and source herbaria, of specimen images initially examined for this study.

**Image database**	**Location**	**Herbaria coverage**
Herbaria@home	http://herbariaunited.org/atHome/	ABS, BIRM, SLBI, DBC, PTH
Digital specimen images at the Herbarium Berolinense.	http://ww2.bgbm.org/herbarium/	B
Appalachian State University	http://vascularflora.appstate.edu/vps	BOON
California Academy of Sciences, botany collection CAS-BOT	http://collections.calacademy.org/bot/	CAS-DS
George Safford Torrey Herbarium (CONN) database	http://bgbaseserver.eeb.uconn.edu/databasesimple.html	CONN
Royal Botanic Garden Edinburgh herbarium catalogue	http://elmer.rbge.org.uk/bgbase/vherb/bgbasevherb.php	E
Botany collections database	http://emuweb.fieldmuseum.org/botany/detailed.php	F
Fairchild Tropical Garden, virtual herbarium	http://www.virtualherbarium.org/vh/db/main.php	FTG
Geneva Herbaria Catalogue	http://www.ville-ge.ch/musinfo/bd/cjb/chg/?lang=en	G, G-DC, G-BOIS, G-BU
Kew Herbarium catalogue	http://apps.kew.org/herbcat/gotoHomePage.do	K
Virtual guide to the main collections of the LE Herbarium	http://www.mobot.org/MOBOT/Research/LEguide/index.html	LE
The Linnean collections	http://linnean-online.org/view/type/specimen/	LINN, LINN-HS
The Herbarium of Louisiana State University, image gallery	http://www.herbarium.lsu.edu/	LSU
Tropicos Image Search	http://www.tropicos.org/ImageSearch.aspx	MO
US National Herbarium: vascular plant types	http://www.usna.usda.gov/Research/Herbarium/BotType.html	NA
C. V, Starr virtual herbarium	http://sciweb.nybg.org/science2/vii2.asp	NY
Sonnerat – Muséum National d’Histoire Naturelle (Paris, France) Spécimens d’herbier	http://coldb.mnhn.fr/colweb/form.do?model=SONNERAT.wwwsonnerat.wwwsonnerat.wwwsonnerat	P
Consortium of Pacific Northwest Herbaria	http://www.pnwherbaria.org/	SOC, WWB
Herbarium, Taiwan Forestry Research Institute, specimen data search	http://taif.tfri.gov.tw/search.php?l=Eng	TAIF
Alabama Plant Atlas	http://www.floraofalabama.org/	TROY
Type specimens in UC and JEPS that have high-resolution images	http://ucjeps.berkeley.edu/db/types/imaged_types.html	UC-JEPS
Databased vascular plant types at UC/JEPS	http://ucjeps.berkeley.edu/db/types/types_table.html	UC-JEPS
US Herbarium, type register search	http://collections.mnh.si.edu/search/botany/?ti=3	US
Atlas of Florida vascular plants, herbarium specimen search	http://florida.plantatlas.usf.edu/specimen.aspx	USF
Virtual Herbaria Austria	http://herbarium.univie.ac.at/database/search.php	W, WU, KUFS, HAL

## Appendix II

**Specimens examined** of *Convolvulus binghamiae* Greene, *Convolvulus limnophilus* Greene, and *Calystegia sepium* L.

**Original, and possibly original material of *Convolvulus binghamiae* Greene. USA. California. Santa Barbara County:** Santa Barbara, “Burton’s Mound” (acc. to the protologue), Aug, 1886 (fl*), Mrs. R.F. Bingham s.n*. (lectotype UC [UC-335392!, originally in the Lemmon Herbarium]; isolectotype F [not seen], according to Brummitt et al. 2012, photos of a “presumed duplicate” are at K and RSA); same locality, July, 1886 (fl, buds) *E.L. Greene s.n*. (NDG [NDG-66274] image; NDG [NDG-66276] image; NDG [NDG-66275] image [no collection month given on specimen]); Santa Barbara, June, 1887, *Mrs. R.F. Bingham 573* (possible original material NA [NA-0026310] image) (http://www.usna.usda.gov/graphics/usna/Research/Herbarium/Specimens/0026310.jpg ). *Mrs. R.F. Bingham 573* is labeled ”type”, but more study is needed. It was collected the same month and year the description was published. Greene states material used in his description was collected in 1886. Since both Bingham and Greene cultivated native morning glories, and Greene included details based on observations of living plants (e.g. the number of days that flowers persisted). It is possible that *Mrs. R.F. Bingham 573* represents a collection of living material that Greene used in his description.

**Original and probably original material of *Convolvulus limnophilus* Greene. USA. California. County unknown:** Suisun Bay, Aug 1883, *E.L. Greene s.n*. (probable syntype NDG [NDG-66290] image); “Gxxx Station” [partly illegible], 1883, *E.L. Greene* (probable syntype NDG [NDG-66293] image).

**Specimens examined of *Calystegia sepium* L. USA. California. Contra Costa County:** levee along Bethel Island, 5 Sep 1954, *I.L. Wiggins 13122*, *13123* (DS); Otto’s Black Bass Resort, 8 Jul 1949, *M.A. Nobs & S.G. Smith 941* (DS). **Los Angeles County:** near Los Angeles, May–June 1904, *Pupils of Los Angeles High School s.n*. (UC); SE of Huntington Beach, 15 Jun 1932, *L.M. Booth 1192* (POM). Riveria [Rivera], 1 May 1902, *A. Davidson 1892* [mixed sheet with *Calystegia felix*] (RSA). **Mariposa County:** Martinez, 30 Jul 1893, *J.W. Congdon s.n*. (CAS). **Orange County:** East of Huntington Beach, 5 Aug 1932, *L.M. Booth 1359* (UC); Bolsa Chica, 28 Jun 1932, *L.M. Booth 1214* (POM); Wintersberg, 26 Sept 1926, *Peirson 7086* (UC, RSA). **Riverside County:** Hidden Valley Wildlife Refuge, 28 May 1989, *M. Braun 39* (UCR); Springbrook, Fairmount Park, Riverside, *S.B. Parish 916, 4612, 5334* (DS), *6435* (CAS); same location, Sep 1921, *E. Smith s.n*. (CAS); same location, 31 May 2004, *Clarke s.n*. (UCR); same location, Aug 1901, *Hall s.n*. (UC); same location, 10 Jun 1952, *J.C. Roos 5763* (UCR); same location, 14 Sep 1999, *M.C. Provance & S. Boyd 1780* (UCR, CAS, RSA, SD, UCD); same location, 29 May 2013, *M.C. Provance & R. Richmeier s.n*. (UCR). **Sacramento County:** Sacramento Valley, 1838–1842, *Wilkes 1365* (US image). **San Bernardino County:** San Bernardino, no date, *Wright 1727* (NDG image); two miles SE of San Bernardino, 17 Jul 1924, *P.A. Munz 8690* (UCR); China Ranch, 7 Aug 1950, *J.C. & A.R. Roos 4919* (RSA); San Bernardino Valley, 7 Sep 1907, *S.B. Parish 6435* (CAS); San Bernardino, Jul 1881, *S.B. & W.F. Parish 916* (DS); E St. Swamp, *S.B. Parish 5334* (DS [2 sheets]); Chino, 16 Jul 1908, *I.J. Condit s.n*. (UC). **San Joaquin County:** Middle River, 22 Aug 1978, *B. Atwater 52* (CAS); Old River, 16 Aug 1978, *B. Atwater 37 (*CAS); Holt, 21 Aug 1946, *H.L. Mason & V. Grant 13067* (CAS). **Solano County:** Collinsville, Sep 1921, *E. Smith s.n*. (CAS); Suisun Marshes, 15 Oct 1905, *W.R. Dudley s.n*. (DS); same location, 15 Oct 1905, *W.R. Dudley s.n*. (DS); same location, 28 Oct 1938, *J.T. Howell 14594* (CAS); same location, 28 Aug 1920, *V. Jones s.n*. (CAS); same location, Jul 1913, *A. Eastwood 3451* (CAS).

## Appendix III

Herbarium specimens examined of the *Calystegia* species included in Table 1.

**Specimens examined. *Calystegia occidentalis* ssp.* fulcrata*. USA. California. Los Angeles County:** Big John Flat, 21 Jun 1999, *Swinney 7365* (UCR); Granite Mountain vicinity, 24 July 1991, *T. Ross & S. Boyd 5602* (UCR). **San Bernardino County:** Fredalba, 22 Jul 1902, *Abrams 2780* (E image, P image); N end of Fawnskin, at base of Delamar Mountain, 2141 m, 14 Jul 1993, *S.D. White 1680* (UCR); San Bernardino Mtns., 1880, *J.C. Nevins 417* (P image), Arrowhead Hot Springs and immediate vicinity, 579 m, *A.C. Sanders 13805* (UCR). **Sonoma County:** near Sonoma [according to R. K. Brummitt], *J.M. Bigelow s.n*. (K image, NY image, PH image). **Tuolumne County:** Twain-Harte, *A. Eastwood & J.T. Howell 8618* (CAS image). ***Calystegia occidentalis* ssp.* occidentalis*. USA. California. County unknown:** near San Francisco, date not known, *H. Gibbons* (GH54299 image). **Amador County:** New York Falls, 1500 ft., 3 Jul 1892, *G. Hansen 79* (E image). **Butte County:** no further location provided, 1848, *M. Hartweg 1862* (P image). **Madera County:** Chowchilla, June 1885, *J.W. Congdon s.n*. (P image). **Napa County:** S. of Lake Berryessa, 15 May 1971, *M.J. Minabe 70* (LSU). **Placer County:** Forest Route 13, 1.8 mi south of junction with Forest Hill Rd., 28 Jul 2010, *G. Helmkamp 16643* (UCR); 0.2 mi north of Forest Hill Rd. on unnamed road to Clementine Lake, 14 Jun 2006, *G. & E.A. Helmkamp 10706* (UCR). **Plumas County:** Meadow Valley, 14 Jul 1963, *B. Horn 630714 #10* (UCR). **Shasta County:** Anderson, 20 April 1968, *G.C. Strausbaugh 29* (WWB image); near Middle Creek Station, 3 Jun 1905, *A.A. Heller 7955* [*4955* on label apparently in error] (E image). **Siskiyou County:** Little North Fork Public Camp, 1997 ft., *C. Miller 56* (SOC image); Yreka, 8 Jun 1905, *A.A. Heller 7997* (E image). **Oregon. Douglas County:** Mt. Nebo, Roseburg, 900 ft., 22 May 1967, *Godfrey 49* (SOC image). **Jackson County:** Jackson City, 22 May 1981, *F.A. Lang 1412* (SOC image). **Lake County:** Mountains near Lakeview, 19 Aug 1901, *W.C. Cusick 2771* (E image). ***Calystegia peirsonii*. USA. California. Los Angeles County:** Rock Creek, 27 May 1923, *F.W. Peirson 3537* (CAS image); same location, 21 May 1923, *Peirson 7301* (UC image); near Palmdale, 9–24 May 1896, *J.B. Davy 2304* (UC image). Bouquet *C. felix* Road east of the reservoir, 20 Jun 1979, *T. Krantz s.n*. (UCR); Castaic Mesa, 28 Apr 2003, *A.C. Sanders 26128* (UCR); Mt. Gleason, 20 Aug 1992, *O. Mistretta 761* (UCR); Newhall Ranch, 19 Jun 2002, *A.C. Sanders & M. Elvin 25200* (UCR). ***Calystegia subacaulis* ssp. *episcopalis*. USA. California.** Acad. du San Francisco, *Anonymous* s.n. (P4492495 image). **San Luis Obispo County:** Cambria, 28 Apr 1926, *A. Eastwood 13641* (K image, CAS image). **San Mateo County:** Woodside, 13 May 1932, *L.S. Rose 32248a* (P image). Above Crystal Springs on the Half Moon Bay road, 28 May 1907, *Heller 8557* (P image). S**onoma County:** Adobe Canyon, just NW of Sonoma, 1200 ft., 24 May 1996, *F. Bowcutt 2163* (UCR).
